# Immunohistochemical categorisation of ductal carcinoma *in situ* of the breast

**DOI:** 10.1038/sj.bjc.6604112

**Published:** 2007-11-27

**Authors:** P Meijnen, J L Peterse, N Antonini, E J Th Rutgers, M J van de Vijver

**Affiliations:** 1 Department of Surgery, The Netherlands Cancer Institute – Antoni van Leeuwenhoek Hospital, Plesmanlaan 121, Amsterdam 1066 CX, The Netherlands; 2 Department of Pathology, The Netherlands Cancer Institute – Antoni van Leeuwenhoek Hospital, Plesmanlaan 121, Amsterdam 1066 CX, The Netherlands; 3 Department of Biometrics, The Netherlands Cancer Institute – Antoni van Leeuwenhoek Hospital, Plesmanlaan 121, Amsterdam 1066 CX, The Netherlands

**Keywords:** ductal carcinoma *in situ* (DCIS), immunohistochemistry, tissue microarray, tumour classification

## Abstract

The aim of this study is to analyse whether immunohistochemistry (IHC) applying a broad set of markers could be used to categorise ductal carcinoma *in situ* (DCIS) of the breast in distinct subgroups corresponding to the recently defined molecular categories of invasive carcinoma. Immunohistochemistry of pure DCIS cases constructed in tissue arrays was performed with 16 markers (oestrogen receptor (ER), progesterone receptor (PR), androgen receptor (AR), Bcl-2, p53, Her2, insulin-like growth factor receptor, E-cadherin, epithelial membrane antigen (EMA), CA125, keratins 5/6, 14, 19, epidermal growth factor receptor, S100, and CD31). Results in 163 cases were analysed by unsupervised hierarchical clustering. Histological classification was performed by review of whole tissue sections and identified 36 well-, 55 intermediately, and 72 poorly differentiated DCISs. Unsupervised hierarchical cluster analysis categorised DCIS into two major groups that could be further subdivided into subgroups based on the expression of six markers (ER, PR, AR, Bcl-2, p53, and Her2). In the major predominantly ER/Bcl-2-positive (luminal) group, three subgroups (AR-positive (*n*=33), AR-negative (*n*=40), and mixed (*n*=34)) could be identified and included 34 well-differentiated DCISs. Within the major predominantly ER/Bcl-2-negative (nonluminal) group, a Her2-positive subgroup (*n*=34) was characterised by 31 poorly differentiated lesions. Eight triple-negative lesions, including one positive for keratin 5/6 and two positive for p53, were encountered. Intermediately differentiated DCIS shared a comparable IHC staining pattern with well-differentiated DCIS that was distinct from poorly differentiated DCIS (*P*<0.001). Ductal carcinoma *in situ* could be categorised by IHC into two major groups and five subgroups using six markers. Morphologically, intermediately differentiated DCIS seems to have more biological similarities with well-differentiated lesions as compared to poorly differentiated lesions.

Breast cancer encompasses a heterogeneous group of tumours, which vary in morphology, clinical presentation, and behaviour. Traditionally, breast cancers are morphologically typed according to the World Health Organization (WHO) guidelines. The latest classification recognises at least 30 different invasive tumour types (World Health Organization Classification of Tumours, 2003). There is no consensus about the classification of the noninvasive precursor of breast carcinoma, ductal carcinoma *in situ* (DCIS). As in other areas of pathology, a three-tier system is most often used, based on growth pattern and cytonuclear criteria, and dividing DCIS into well-, intermediately, and poorly differentiated subtypes (Holland *et al*, 1994). In prospective studies, this classification has proven value in risk assessment of recurrence after breast-conserving treatment and progression into invasive carcinoma (Bijker *et al*, 2006). However, inter- and intraobserver variability is a problem inherent to morphologic tumour classification and grading (Sloane *et al*, 1998); moreover, heterogeneity within DCIS lesions is not uncommon, resulting in variation in grade (Ma *et al*, 2003). Perou *et al* (2000) suggested a categorisation of invasive breast cancers based on genetic profiles into oestrogen receptor (ER)-positive (luminal A and B) and ER-negative (nonluminal) subtypes with a further subdivision of the ER-negative types into Her2-positive and basal-like subtypes. Luminal A tumours differ from luminal B tumours by a higher expression of ER-related genes and lower expression of proliferation-associated genes. It was possible to make the same categorisation by immunohistochemistry (IHC) using markers aimed at luminal, Her2, and basal-like features (Makretsov *et al*, 2004). The objective of this study is to classify DCIS by marker expression to improve the current morphological classifications and gain insight into the biology underlying the heterogeneity in DCIS. Therefore, tissue microarrays were constructed from a series of pure DCISs, a large set of markers were used for IHC, and unsupervised hierarchical cluster analysis was performed to evaluate results; clustering was correlated with morphologic grade of DCIS as assessed on whole tumour slides.

## MATERIALS AND METHODS

Tissue microarray sections were constructed taking three 0.6-mm tissue cores per case, from formalin-fixed, paraffin-embedded tumour blocks with pure DCIS of 238 patients using a tissue-arraying instrument (Beecher Instruments, Silver Spring, MD, USA). Immunohistochemistry was performed on an automated stainer after pretreatment in the autoclave in citrate buffer at pH 6.0 according to standardised protocols for the different antibodies at prescribed dilutions (see Table 1). Sixteen markers were used including ER, progesterone receptor (PR), androgen receptor (AR), Her2, Bcl-2, p53, E-cadherin, epidermal growth factor receptor, insulin-like growth factor receptor, CD31, keratin 5/6, keratin 14, keratin 19, S100, epithelial membrane antigen (EMA), and CA125. The selection of antibodies was based on recent investigations in gene signature profiles in invasive breast cancer, suitability for DCIS, and availability. Staining results were semi-quantitatively scored according to the criteria in Table 1. The higher IHC score was considered as a final score in case of a difference between tissue cores. Cutoff points are shown in Table 1 and were directed to detect luminal and nonluminal (sub)groups. All cases were classified as well-, intermediately, and poorly differentiated on the whole tumour slides according to the classification of Holland *et al* (1994); in case of heterogeneity, the highest grade was used for analysis. The distribution of markers and histological grade among (sub)groups was analysed using the *χ*^2^-test or Fisher's exact test. Tests were two-tailed and the significance level was taken 5%. Discriminative markers underwent unsupervised hierarchical clustering analysis with average and complete linkage (Genesis 1.5.0; IGB-TUG, Graz, Austria) to organise tissue microarray score data into meaningful structures, in accordance with the more complex method used for cDNA microarrays (Weigelt *et al*, 2005). The impact of the markers on hierarchical cluster group results was investigated to define a final set of markers for IHC classification. Correlation between markers was determined using the Spearman's correlation coefficient. The agreement in classification of cases based on different hierarchical clustering methods (average linkage *vs* complete linkage) and different IHC classifications were assessed with the *κ*-statistic. A *κ*-value of 0.41–0.6 was considerate moderate agreement, 0.61–0.8 substantial agreement, and more than 0.8 near-perfect agreement. All analyses were performed in SPSS® 11.5 for Windows (SPSS, Chicago, IL, USA).

## RESULTS

Of the 238 DCIS samples, 27 (11%) did not contain tumour and 48 (20%) had incomplete IHC data due to loss of tissue. All analyses were performed on the remaining 163 cases. Median age of these patients was 50 years (range: 28–82 years). Seventy-three per cent of the lesions were screen detected. Histological classification identified 36 (22%) well-, 55 (34%) intermediately, and 72 (44%) poorly differentiated lesions.

### Marker expression in DCIS

Table 2 presents the distribution of the different markers in DCIS. Oestrogen receptor and PR were most frequently present in well- and intermediately differentiated DCIS (*P*<0.001), whereas Her2 expression was most frequently found in poorly differentiated DCIS (*P*<0.001). Also, Bcl-2 and p53 expression was different among grades: well-differentiated DCIS were often Bcl-2 positive and p53 negative compared to poorly differentiated DCIS. Thirty-three out of thirty-six well-differentiated DCISs stained positive for Bcl-2 compared to 26 out of 72 poorly differentiated DCISs (*P*<0.001). Further, all well-differentiated DCISs were p53 negative, while half of the poorly differentiated lesions were p53 positive (*P*<0.001). Moderately differentiated DCIS formed an intermediate group in expression of these markers with the exception of AR. This marker was found positive in 28 intermediately differentiated DCISs compared to 13 well-differentiated and 19 poorly differentiated DCISs (*P*=0.018). The remaining markers showed no statistically significant association with grade of DCIS. The E-cadherin protein could be detected in all DCISs, while markers for EGFR, CD31, keratin 14, S100, and CA125 showed negative staining results in all lesions. insulin-like growth factor receptor, keratin 19, and EMA were found positive in nearly all DCISs except seven DCISs. Five out of these seven DCISs were poorly differentiated. Three poorly differentiated DCISs showed staining for keratin 5/6.

### IHC categorisation by unsupervised hierarchical analysis

**Figure 1:**
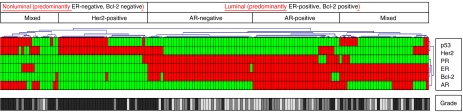
Two-dimensional unsupervised hierarchical cluster diagram (average linkage) of data consisting of 978 genes by 163 ductal carcinoma *in situ* samples. Clustergram of six markers and distribution of histological grade. Each column indicates a single case; each row, a single immunomarker. Green (or light grey), negative immunostaining; red (or dark grey), positive immunostaining; white, well-differentiated lesion; grey, intermediately differentiated lesion; black, poorly differentiated lesion. The dendrogram shows the relatedness of the immunoprofiles of individual cases and suggests two major groups, which are further subdivided into subgroups.

Unsupervised hierarchical clustering analysis was applied to the IHC data set. On the basis of the expression of ER and Bcl-2, the clustergram in Figure 1 shows two major groups: a predominantly ER/Bcl-2-positive (luminal) and a predominantly ER/Bcl-2-negative (nonluminal) group. These two groups can be further subdivided using other marker results. The luminal group demonstrated a completely AR-positive subgroup (*n*=33), a completely AR-negative subgroup (*n*=40), and a mixed subgroup of AR-positive and -negative lesions (*n*=34), while the nonluminal group included a completely Her2-positive cluster (*n*=34). The luminal lesions included 34 (94%) well-differentiated, 46 (84%) intermediately differentiated, and 27 (38%) poorly differentiated DCISs. These poorly differentiated DCISs lesions showed markers positive for ER (all 27), Bcl-2 (*n*=25), PR (*n*=14), Her2 (*n*=12), AR (*n*=9), and p53 (*n*=4).

The nonluminal subgroups had 45 poorly differentiated DCISs and 11 nonpoorly differentiated lesions including nine with intermediately and two with well-differentiated DCISs. These 11 lesions showed a marker pattern that was positive for ER (*n*=2), Bcl-2 (*n*=1), Her2 (*n*=7), AR (*n*=5), and p53 (*n*=2). In total, eight ER-negative, PR-negative, and Her2-negative lesions, including one positive for keratin 5/6 and two positive for p53, were found. Table 3 shows the comparison of the distribution of histological grade among the identified subgroups. Intermediately differentiated DCIS significantly more often shared IHC features with well-differentiated DCIS than with poorly differentiated DCIS (*P*<0.001).

### Reproducibility of cluster groups

For the assessment of variation in clustering results when using different hierarchical clustering methods, unsupervised hierarchical clustering by complete linkage was performed on the classification set of six markers. The concordance between designation of individual cases to one of the subgroups using average linkage *vs* complete linkage showed a near-perfect agreement (*κ*=0.876), with 16 mismatches out of 163 paired cases.

### Comparison with IHC categorisation based on genetic profiles

A comparison of our results with the earlier findings from Perou *et al* (2000) based on genetic profiles of invasive breast carcinoma is shown in Table 4. A classification of DCIS lesions into luminal A, luminal B, Her2, and basal-like subtypes was performed on the staining results of three markers (ER, PR, and Her2) and was compared with the findings of the present study. Both classifications showed a moderate agreement (*κ*=0.411) mainly caused by the differentiation of the luminal types into A and B. If both the luminal types are considered as one group, the classifications demonstrated a substantial agreement (*κ*=0.649). Nearly complete agreement is shown for the Her2-positive and basal-like type lesions. Mixed lesions from the Perou *et al* (2000) classification frequently showed ER and Bcl-2 marker expression.

## DISCUSSION

The traditional histological classification of invasive breast cancer tumours has been debated by results from gene expression arrays leading to the molecular categorisation of breast cancer into luminal and nonluminal tumours (Perou *et al*, 2000). As invasive ductal breast cancers develop via the noninvasive precursor DCIS, these lesions may be categorised in the same way. Most similarities were found among the different stages of breast tumour progression, and it was suggested that gene expression alterations conferring the potential for invasive growth are already present in the preinvasive stadium of breast cancer (Ma *et al*, 2003). It has been shown that the molecular subgroups of invasive carcinoma can be distinguished using a set of IHC markers (Makretsov *et al*, 2004). In addition, response to treatment is subgroup dependent (Carey *et al*, 2007). Using IHC, our analysis focused on the identification of subgroups in pure DCIS, to improve insight into the different pathways of tumour development, and to produce a classification of DCIS based on marker expression. Sixteen markers were selected to distinguish luminal and nonluminal cell differentiation, or because of their reported value to differentiate DCIS. Unsupervised hierarchical analysis, like in the evaluation of gene expression arrays, was performed to categorise DCIS. Ten markers were either positive or negative in nearly all DCISs and therefore not useful for classification.

Oestrogen receptor, PR, AR, Her2, Bcl-2, and p53 were used for IHC classification of DCIS. Oestrogen receptor, PR, and AR were positive in 68, 46 and 37% of the patients in our series, respectively. Others found ER, PR, and AR expression in 54–73, 49–61, and 33–44%, respectively (Selim *et al*, 2002; Baqai and Shousha, 2003; Barnes *et al*, 2005; Rody *et al*, 2005). We further found that well- and intermediately differentiated DCISs were predominantly ER positive and PR positive, while poorly differentiated DCIS usually lacked steroid receptor expression and was correlated with Her2 overexpression. This finding became further evident by the unsupervised hierarchical clustering results that clearly divided DCIS into luminal and nonluminal lesions. The luminal-type DCIS was further divided into an AR-positive and AR-negative subtype.

Bcl-2, involved in apoptosis, was present in 64% of all DCISs, while p53 was expressed in 26% of the cases in our series. These findings are in correspondence with results from others who reported Bcl-2 and p53 expression in 76 and 24% of DCIS cases, respectively (Siziopikou *et al*, 1996). The Bcl-2-positive/p53-negative phenotype, which is similar to normal epithelium and benign lesions, was observed in 95 cases originating from the luminal clusters. This phenotype might reflect a more favourable group of lesions.

Androgen receptor expression was most frequently seen in intermediately and well-differentiated DCIS (*P*=0.018) in our series of patients. Not many studies investigated AR in DCIS. Moinfar *et al* (2003) reported a higher rate of AR expression in especially low-grade DCIS as opposed to high-grade DCIS, although others did not find a correlation between AR expression and grade (Selim *et al*, 2002). Androgen receptor-positive breast cancer patients have prolonged survival and a better response to hormonal treatment than AR-negative patients.

Within the nonluminal type, a Her2-positive/ER-negative subtype with 91% poorly differentiated DCISs could be identified. Her2 is known to be amplified and/or overexpressed in invasive breast cancer in 10–30% of cases and associated with poor outcome (Ravdin and Chamness, 1995; Tsuda, 2001). The absence of Her2 overexpression in normal ducts and atypical ductal hyperplasia, and the frequent of Her2 amplification found in DCIS suggests that Her2 alterations are an early event in the pathway of development of Her2-positive invasive carcinomas. In our study, 39% of the cases were positive for Her2. The higher frequency of Her2-positive lesions in DCIS compared with invasive breast cancer has been argued to occur due to loss of expression; however, it might indicate that in the breast cancer progression model, there may be lesions that do not frequently evolve into invasive breast cancers, including Her2-positive DCIS lesions. Moreover, the mammographic detection of poorly differentiated Her2-positive DCIS often occurs at an early stage due to the conspicuous microcalcifications.

Basal-like carcinomas have been identified in gene expression profiling studies as a subtype of invasive breast cancer. These lesions are ER negative, PR negative, and Her2 negative (triple negative). We found eight (5%) triple-negative lesions. Four of them were poorly differentiated. Bryan *et al* (2006) studied 66 cases of high nuclear grade DCIS to determine the frequency of the triple-negative phenotype and showed that only four cases (6%) exhibited the triple-negative phenotype. In contrast with our results, they found EGFR expressed in all four triple-negative lesions and also in a selection of nontriple-negative lesions, while we found negative staining in all lesions. In addition, they observed more frequent expression of keratins 5/6 and 14 compared with our series. This could be a result of the interobserver variability, since our cutoff point was more than 10% strong membraneous staining, while Bryan *et al* (2006) considered any staining as positive. Recently, (Livasy *et al* (2007) found 8% basal-like subtypes in a population-based series of 245 patients. Given that invasive breast cancers typically share immunophenotypic features with the DCIS lesion from which they arise, these findings corroborate the possibility that the triple-negative DCIS lesions represent a precursor lesion to invasive basal-like carcinomas. In these (medullary-like and metaplastic) carcinomas, *in situ* components are usually minor or absent, suggesting a rapid progression from *in situ* to the invasive stage. This is in keeping with the absence of basal-like *in situ* lesions in preventive mastectomy specimens of BRCA1 carriers, which are prone to develop basal-like tumours (Hoogerbrugge *et al*, 2003).

Clustering analysis showed that the well-differentiated DCIS and intermediately differentiated DCIS share IHC features among the different clusters. It seems that intermediately differentiated DCIS shows more resemblance with well-differentiated DCIS as compared with poorly differentiated DCIS. A recent study from our institute investigating classification of DCIS by gene expression profiling confirms this finding and identified luminal, Her2-, and basal-like tumours in a series of 40 DCIS lesions (Hannemann *et al*, 2006). A classification of DCIS by IHC might identify identical groups of luminal and nonluminal tumours, which can be further subdivided, reflecting the heterogeneous nature of DCIS. Therefore, IHC can assist in objectivation of variations in morphologic tumour classification of DCIS.

## Figures and Tables

**Table 1:** List of antibodies and tissue microarray scoring criteria

	**Marker**	**Clone**	**Source**	**Dilution**	**Staining pattern**	**Cutoff point**
1	ER	1D5+6F11	Neomarker	1 : 50	Nuclear	Any +
2	PR	PR-1	ImmunoVision	1 : 500	Nuclear	Any +
3	AR	AR441	Neomarker	1 : 400	Nuclear	Strong >10%
4	Her2	3B5	Neomarker	1 : 80 000	Membranous	Strong >10%
5	Bcl-2	124	DAKO	1 : 400	Cytoplasmic	Weak >10%
6	p53	DO-7	DAKO	1 : 1000	Nuclear	>25%
7	E-cadherin	HECD-1	Intermedico/Zymed	1 : 2500	Membranous	Weak >10%
8	EGFR	111.6	Neomarker	1 : 200	Membranous	Strong >10%
9	IGFR	24–31	Neomarker	1 : 100	Cytoplasmic and membranous	Weak >10%
10	CD31	JC/70A	DAKO	1 : 50	Cytoplasmic	Any +
11	Keratin 5/6	D5/16B4	DAKO	1 : 200	Cytoplasmic	Any +
12	Keratin 14	LL002	Neomarker	1 : 200	Cytoplasmic	Any +
13	Keratin 19	RB-9021^a^	Neomarker	1 : 200	Cytoplasmic	Weak >10%
14	S100	Z0311^a^	DAKO	1 : 4000	Cytoplasmic	Any +
15	EMA	E29	DAKO	1 : 1000	Cytoplasmic and membranous	Weak >10%
16	CA125	BGX324A	Biogenex	1 : 80	Membranous	Weak >10%

AR=androgen receptor; EGFR=epidermal growth factor receptor; EMA=epithelial membrane antigen; ER=oestrogen receptor; IGFR=insulin-like growth factor receptor; PR=progesterone receptor.

DAKO, Glostrup, Denmark; Neomarker, Fremont, CA, USA; Intermedico/Zymed, San Francisco, CA, USA; ImmunoVision, Springdale, AR, USA; and Biogenex, San Ramon, CA, USA.

a Catalogue number.

**Table 2:** Expression of markers in well-, intermediately, and poorly differentiated DCIS

		**Histological grade (%)**			
**Type and marker**	**Total (%)**	**Well, *n*=36 (22)**	**Intermediate, *n*=55 (34)**	**Poor, *n*=72 (44)**	***P***
ER	111 (68)	34 (94)	47 (86)	30 (42)	<0.001
PR	75 (46)	26 (72)	33 (60)	16 (22)	<0.001
AR	60 (37)	13 (36)	28 (51)	19 (26)	0.018
Her2	64 (39)	1 (3)	11 (20)	52 (72)	<0.001
Bcl-2	105 (64)	33 (92)	46 (84)	26 (36)	<0.001
p53	42 (26)	0	7 (13)	35 (49)	<0.001
E-cadherin	163 (100)	36 (100)	55 (100)	72 (100)	—
EGFR	0	0	0	0	—
IGFR	157 (96)	35 (97)	54 (98)	68 (94)	0.513
Keratin 5/6	3 (2)	0	0	3 (4)	0.145
Keratin 14	0	0	0	0	—
S100	0	0	0	0	—
Keratin 19	162 (99)	35 (97)	55 (100)	72 (100)	0.170
EMA	162 (99)	36 (100)	55 (100)	71 (99)	0.529
CA125	0	0	0	0	—
CD31	0	0	0	0	—

AR=androgen receptor; DCIS=ductal carcinoma *in situ*; EGFR=epidermal growth factor receptor; EMA=epithelial membrane antigen; ER=oestrogen receptor; IGFR=insulin-like growth factor receptor; PR=progesterone receptor.

Values in parentheses are percentages.

**Table 3:** Distribution of markers and histological grade among cluster group after unsupervised hierarchical clustering analysis with six markers (ER, PR, AR, Bcl-2, Her2, and p53)

	**Distribution of markers and grade by group and subgroup**								
	**Luminal group**				**Nonluminal group**			***P* a**	
	**Total**	**AR-positive subgroup**	**AR-negative subgroup**	**Mixed subgroup**	**Total**	**Her2-positive subgroup**	**Mixed subgroup**	**Two groups**	**Subgroups**
Number of patients	107 (66)	33 (20)	40 (25)	34 (21)	56 (34)	34 (21)	22 (14)		
									
*Markers*									
ER	106 (99)	33 (100)	39 (98)	34 (100)	5 (9)	0	5 (23)	<0.001	<0.001
PR	73 (68)	30 (91)	40 (100)	3 (9)	2 (4)	2 (6)	0	<0.001	<0.001
AR	45 (42)	33 (100)	0	12 (35)	15 (27)	0	15 (68)	0.055	<0.001
Bcl-2	103 (96)	30 (91)	39 (98)	34 (100)	2 (4)	1 (3)	1 (5)	<0.001	<0.001
Her2	18 (17)	6 (18)	6 (15)	6 (18)	46 (82)	34 (100)	12 (55)	<0.001	<0.001
p53	9 (8)	0	0	9 (27)	33 (59)	20 (59)	13 (59)	<0.001	<0.001
									
*Grade*									
Well	34 (32)	11 (33)	15 (38)	8 (24)	2 (4)	1 (3)	1 (5)	0.190^b^	0.474^b^
Intermediate	46 (43)	17 (52)	16 (40)	13 (38)	9 (16)	2 (6)	7 (32)		
Poor	27 (25)	5 (15)	9 (23)	13 (38)	45 (80)	31 (91)	14 (64)	<0.001^c^	<0.001^c^
									

AR=androgen receptor; DCIS=ductal carcinoma *in situ*; ER=oestrogen receptor; PR=progesterone receptor.

Values in parentheses are percentages.

a *χ*^2^-test.

b Intermediately differentiated DCIS *vs* well-differentiated DCIS.

c Intermediately differentiated DCIS *vs* poorly differentiated DCIS.

**Table 4:** Comparison of IHC classification of DCIS based on ER, PR, and Her2 expression (in analogy of Perou *et al*, 2000) *vs* IHC classification based on ER, BCL-2, AR, and Her2 expression (present study) and relation with histological grade

**IHC classification based on ER, BCL-2, AR, and Her2 expression**	**Luminal A (ER+, PR+, Her2−)**	**Luminal B (ER+, Her2+)**	**Her2 (ER−, Her2+)**	**Basal-like (ER−, PR−, Her2−)**	**Mixed**	**Total**
Luminal, AR+ (well/intermediate/poor)	27 (10/15/2)	6 (0/4/2)	0	0	9 (2/3/4)	42 (12/22/8)
Luminal, AR− (well/intermediate/poor)	34 (15/15/4)	10 (0/1/9)	0	0	16 (6/6/4)	60 (21/22/17)
Nonluminal, Her2+ (well/intermediate/poor)	0	0	42 (1/4/37)	0	0	42 (1/4/37)
Nonluminal, Her2− (well/intermediate/poor)	0	0	0	7 (1/2/4)	0	7 (1/2/4)
Mixed (well/intermediate/poor)	2 (1/1/0)	5 (0/2/3)	1 (0/0/1)	1 (0/1/0)	3 (0/1/2)	12 (1/5/6)
Total (well/intermediate/poor)	63 (26/31/6)	21 (0/7/14)	43 (1/4/38)	8 (1/3/4)	28 (8/10/10)	163 (36/55/72)

AR=androgen receptor; ER=oestrogen receptor; IHC=immunohistochemistry; PR=progesterone receptor.
